# A hybrid method for generation of attenuation map for MR-based attenuation correction of PET data in prostate PET/MR imaging

**DOI:** 10.1186/2197-7364-1-S1-A77

**Published:** 2014-07-29

**Authors:** Mehdi Shirin Shandiz, Mohammad Hadi Arabi, Pardis Ghafarian, Mehrdad Bakhshayesh Karam, Hamidreza Saligheh Rad, Mohammad Reza Ay

**Affiliations:** Department of Medical Physics and Biomedical Engineering and Research Center for Molecular and Cellular Imaging, Tehran University of Medical Sciences, Tehran, Iran; Chronic Respiratory Disease Research Center, NRITLD and PET/CT and Cyclotron Center, Masih Daneshvari Hospital, Shahid Beheshti University of Medical Sciences, Tehran, Iran

Recently introduced PET/MRI scanners present significant advantages in comparison with PET/CT. However, the lack of accurate method for generation of µmap from MR images for implementation of MRAC is hampering further development. In this study, we present a new method including short echo-time pulse sequence to detect bone signal along with a robust and automatic image segmentation method base on FCM, active contouring and shape analysis to provide a three-classes µmap. The proposed imaging protocol implemented on a 1.5T Avanto scanner. The acquisition parameters were 1.11 msec and 20 msec for TE and TR, respectively, with FA=20. The image-processing protocol includes five major steps: (I) intensity-inhomogeneity correction using non parametric method, which is an essential step for removing bias field; (II) separation of bone and air from other areas using active contouring based on gradient vector method; (III) FCM in order to segment the image into two clusters, one cluster is bone and air and another cluster is soft tissue; (IV) separation of bone and air areas using shape analysis; (V) generation of µmap. The accuracy of the proposed segmentation method was validated using comparison against manual segmentation. The corresponding segmentation images and generated µmap prove the validity of algorithm. Quantitative analysis on accuracy, sensitivity and specificity for 15 segmented images in comparison with manual segmentation performed by an expert radiologist. The proposed strategy shows that the three tissue class µmap can be successfully generated from MR images in pelvis region using STE pulse sequence following by the image processing steps. The method can be a potential alternative to UTE-based attenuation correction. The algorithm is still under development and will be validated in more details based on comparison with the µmaps generate from CT images. This study has been developed particularly for improving the accuracy of MRAC in prostate imaging.

